# Feather corticosterone does not correlate with environmental stressors or body condition in an endangered waterbird

**DOI:** 10.1093/conphys/coaa125

**Published:** 2020-12-30

**Authors:** Brenna M G Gormally, Charles B van Rees, Emily Bowers, J Michael Reed, L Michael Romero

**Affiliations:** 1 Department of Biology, Tufts University, Medford, MA 02155, USA; 2 Current Address: Schmid College of Science and Technology, Chapman University, Orange, CA, USA; 3 Current Address: Flathead Lake Biological Station and Department of Wildlife Biology, University of Montana, Polson, MT, USA

**Keywords:** Bioindicator, conservation, feather corticosterone, fragmentation, glucocorticoids, Hawaiian gallinules, urbanization, wetlands

## Abstract

Physiological metrics are becoming popular tools for assessing individual condition and population health to inform wildlife management and conservation decisions. Corticosterone assays can provide information on how animals cope with individual and habitat-level stressors, and the recent development of feather assays is an exciting innovation that could yield important insights for conservation of wild birds. Due to the widespread enthusiasm for feather corticosterone as a potential bioindicator, studies are needed to assess the ability of this technique to detect meaningful differences in physiological stress across a variety of stressor types and intensities. We examined feather corticosterone from 144 individuals among the 13 known breeding populations of Hawaiian gallinule (*Gallinula galeata sandvicensis*), an endangered waterbird, on the island of O‘ahu. These ecologically independent subpopulations are known to have low genetic connectivity and movement rates and differ largely across a number of important conditions, including level of predator management, human disturbance, proximity to urban development and conspecific population density. This system is well suited for assessing the performance of feather corticosterone as a bioindicator of different known habitat-level threats common to this and many other conservation-reliant species. We found no statistically significant relationship between feather corticosterone and level of predator control, level of human disturbance, gallinule population density, percent urban cover or body condition across all sites despite the substantial difference in stressor magnitude in our dataset. We did find that gallinules in habitats with larger population densities were in worse body condition. These findings suggest that feather corticosterone is not a consistent indicator of anthropogenic impacts on populations. Furthermore, they suggest that feather corticosterone may be a poor bioindicator of known habitat-level threats for Hawaiian gallinules and that it should be used with caution in other avian taxa of conservation concern.

## Teaser

Feather corticosterone is a popular conservation physiology technique to assess stress in wild populations. We show that it does not correlate with habitat (human disturbance, urbanization, predation, density) or individual (body condition) level stressors in the Hawaiian gallinule, an endangered waterbird that experiences a robust ‘stressor gradient’ across its range.

## Introduction

In recent years, wildlife biologists and conservation scientists have rapidly expanded the use of physiological tools to assess population and individual health for animal species of conservation concern. The goal of conservation physiology (Carey, 2005; Tracy *et al.*, 2006; Wikelski and Cooke, 2006; Ellis *et al.*, 2012) is to assess physiological parameters that are relevant to population viability in order to provide rapid and accurate information to guide conservation and management. Among the most popular and widely used physiological parameters used in conservation assessment in vertebrates are the stress-related glucocorticoid hormones (e.g. Walker *et al.*, 2005; Madliger *et al.*, 2015; Bergman *et al.*, 2019). Corticosterone (the primary glucocorticoid in birds) is a steroid hormone that is released from the hypothalamic pituitary adrenal/interrenal (HPA/I) axis. In response to acute stressors (lasting minutes to hours), the release of corticosterone is elevated, which leads to the general upregulation of essential and downregulation of nonessential survival systems (reviewed by Sapolsky *et al.*, 2000). At baseline and acute stress-induced levels, corticosterone is crucial for maintaining homeostasis and coping with short-term stressors; however, when chronically activated—either due to prolonged or severe stressors—corticosterone can lead to harmful effects including immunosuppression and hypertension (reviewed by Romero and Wingfield, 2016).

Due to the importance that corticosterone plays in the vertebrate stress response and its sensitivity to longer-term stressors, it has become a popular metric in conservation physiology. The regulation of corticosterone has been shown to be affected by a number of habitat-level stressors including quality (Homan *et al.*, 2003), fragmentation (Brearley *et al.*, 2012), automobile traffic (Monti *et al.*, 2018; Zollinger *et al.*, 2019) and human presence/disturbance (Walker *et al.*, 2006; Monti *et al.*, 2018). Despite the numerous studies linking corticosterone with population-level variables, neither chronic stress (Dickens and Romero, 2013) nor conservation status (Martin *et al.*, 2018) has been shown to consistently and predictably affect corticosterone. A potential reason that such patterns have been missed, if present, is an insufficiently robust gradient of habitat quality among sample sites, and a small number of local population replicates, which limit the statistical power to detect biologically important interactions between habitat and physiological stress.

A recent methodological innovation in stress physiology research is the extraction of corticosterone from bird feathers (Bortolotti *et al.*, 2008; Lattin *et al.*, 2011), which yields an estimate of corticosterone levels integrated across the growth period of the feather (reviewed by Romero and Fairhurst, 2016). After completing growth, feathers are no longer attached to the blood supply; however, the deposited hormones remain archived in the structures (Brush, 1978; Sheriff *et al.*, 2011). Thus, the feather is a built-in time capsule of hormone exposure during the period of feather growth. Opportunistic sampling of feathers can provide a window into a bird’s physiology during molt. The ease, rapidity and reduced invasiveness of sample collection in this method have great potential for monitoring and management of individuals and populations of conservation concern. However, testing and validation of this method across a large gradient of habitat and individual variables and a larger number of replicate populations are necessary to gauge the reliability and sensitivity of this potential management tool.

We investigated this potential using the Hawaiian gallinule (‘Alae ‘Ula; *Gallinula galeata sandvicensis*), an endangered subspecies of the common gallinule, which persists only on the islands of Kaua‘i and O‘ahu (US Fish and Wildlife Service, 2011). Though originally found throughout the archipelago, substantial habitat loss and predation by introduced invasive species caused severe population constriction (Schwartz and Schwartz, 1949; Banko, 1987). They are found in highly fragmented freshwater coastal wetlands between which dispersal is very limited (van Rees *et al.*, 2018a, 2018b). Wetland habitat patches span the urban–rural gradient, from highly managed national wildlife refuges with little to no public access, to water hazards in public golf courses and wetlands adjacent to urban sprawl and popular shopping centers. While gallinules are not food limited (DesRochers *et al.*, 2010), there is intense intraspecific competition for space that often leads to violent agonistic interactions (van Rees *et al.*, in press). Hawaiian gallinules are one of several endemic, endangered Hawaiian waterbirds that are considered conservation reliant (Reed *et al.*, 2012; Underwood *et al.*, 2013), requiring continuous management of invasive plants and introduced mammalian predators to maintain viable populations (Underwood *et al.*, 2013). The presence and impacts of invasive mammalian predators are thus a widely accepted threat to Hawaiian gallinule populations, with the possibility of complete reproductive failure where management is absent (US Fish and Wildlife Service, 2011; Underwood *et al.*, 2013). Invasive predator control, human disturbance and gallinule population density vary widely between isolated subpopulations, resulting in a wide variation of conservation-relevant, habitat-level environmental stressors across O‘ahu’s landscape.

Because of their range across diverse habitat types and discrete distribution of replicated, independent habitat patches on O‘ahu, Hawaiian gallinules create a compelling system to examine the effects of different environmental stressors on feather corticosterone. In this study, we collected feather samples from 144 Hawaiian gallinules across 13 different subpopulations with widely different stressor levels across these important habitat characteristics. We hypothesized that hormone concentrations would correlate with individual and habitat-level covariates including conspecific population density, human disturbance (measured as encounter rate with people), degree of urbanization and index of predator density. Specifically, we predicted that corticosterone would be the highest in birds in populations with larger densities, increased interactions with humans and higher predation pressures, as these situations represented more challenging environments for endangered species. These predictions are based on the findings that corticosterone becomes persistently upregulated in more energy-expensive situations, including intraspecific competition (reviewed by Creel *et al.*, 2013) and perceived predation threats (e.g. Clinchy *et al.*, 2004; Zanette *et al.*, 2019).

**Table 1:** Summary of sites where Hawaiian gallinules (HAGA) were sampled in this study on the island of O‘ahu, with accompanying habitat classification

Site	Description	n	Sampling year(s)	Predator control^1^	% urban cover within 1 km	Human visitation^2^ rate/day	HAGA density (birds/ha)
Enchanted Lakes	State wildlife refuge (urban)	16	2015, 2016	1	61.5	1–10	12.67
Hamakua	State wildlife refuge (urban)	19	2015, 2016	1	64.5	>100	11.88
Honouliuli	National wildlife Refuge (urban)	2	2015	0	21.6	<1	2.42
James Campbell	National wildlife refuge (rural)	9	2014, 2015	0	13.4	<1	2.69
Kawainui	State wildlife refuge	6	2016	1	32.0	<1	0.726
Keawawa	Private reserve (urban)	7	2014–2016	1	63.7	51–100	7.83
Klipper	Golf course (military)	15	2016	2	51.6	11–50	23.4
Lotus	Private farm	17	2015, 2016	2	35.3	1–10	13.35
Olomana	Golf course (public)	14	2015, 2016	2	28.2	11–50	4.76
Pouhala	Wildlife sanctuary	9	2016	1	41.6	<1	0.67
Shrimp	Aquaculture farm	4	2016	2	16.4	1–10	2.07
Turtle Bay	Golf course (private)	13	2015	2	17.4	11–50	3.36
Waimea	Botanical garden	13	2014–2016	1	4.0	>100	34.86

^1^Level of predation control was determined via consultation with land managers. 0 was assigned to sites with heavy and constant predator control via active exclusion and/or removal. 1 was assigned to sites with moderate, limited or inconsistent predator control. 2 was assigned to sites with no predator control.

^2^Daily human visitation rates were approximated via field observations over the course of a minimum of 7 days per site.

**Figure 1:**
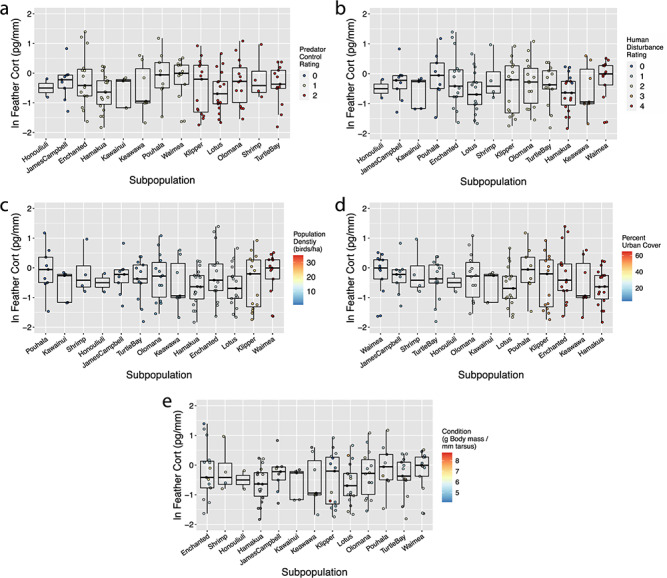
Feather corticosterone in Hawaiian gallinules does not differ across 13 different subpopulations on O‘ahu. Note the x-axis changes between panels and is organized according to the grouping covariate (color). (A) Values of 0, 1 and 2 correspond to sites with active/constant predator control, limited predator control and no predator control, respectively. (B) Sites were assigned a level of human disturbance according to daily visitation rates. Higher values (red) indicate sites with more human disturbance. (C) Higher values (red) indicate sites that have larger densities of gallinules. (D) Higher values (red) indicate sites with more urban cover within 1 km. (E) Higher values (red) indicate birds in better condition (body mass in g/tarsus length in mm). Individuals in grey indicate birds for which condition data were unavailable (*n* = 34). Note that three birds are left off of each panel because the corresponding feather corticosterone values fall outside two standard deviations.

## Materials and methods

### Site selection and habitat characteristics

**Figure 2:**
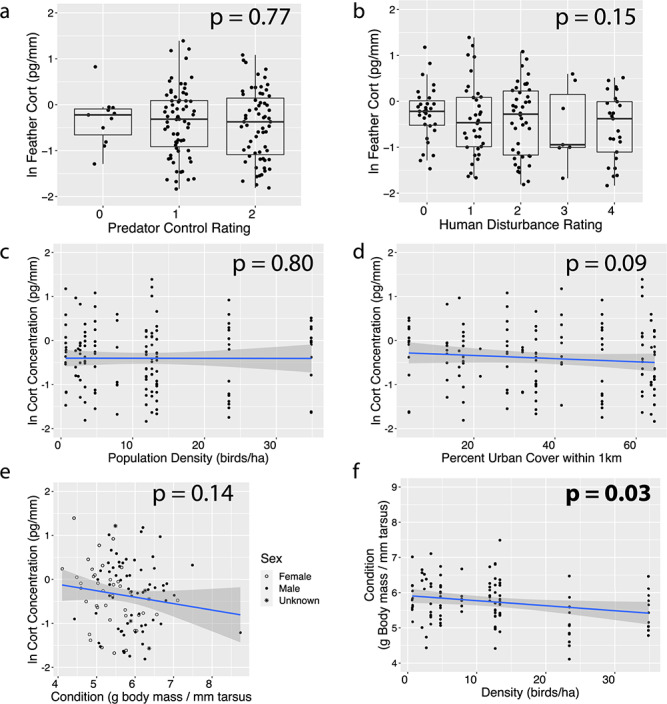
Comparisons between feather corticosterone in Hawaiian gallinules and individual and habitat-level covariates of interest. Feather corticosterone is not correlated with (A) level of predation control, (B) level of human disturbance, (C) density of gallinules, (D) percentage of urban cover within 1 km or (E) body condition (body mass in g/tarsus length in mm). Thirty-six birds have been left off of this panel, 34 did not have associated body condition data and 2 additional birds had feather corticosterone values that fall outside two standard deviations. (F) Birds in populations with greater densities of gallinules tended to be in worse body condition. *P* values are indicated on each panel, with any significant values in bold. Note that three birds are left off of panels A–D because the corresponding feather corticosterone values fall outside two standard deviations.

Over three summers (May–July 2014–2016; +2 samples collected in December), Hawaiian gallinules were captured and sampled at 13 different locations in O‘ahu, Hawaii, USA (for a map depicting all sample locations, see Fig. 2 in van Rees *et al.*, 2018a, and Fig. 1 in van Rees et al., 2018b). The locations differed in levels of urbanization and human visitations and represent all known major breeding areas for the subspecies on the island. Samples were usually captured within the same 6-week period during the summer season, with little to no changes in water levels, mean daily temperatures or precipitation observed throughout the field season (see below for additional remarks on temporal homogeneity in abiotic conditions).

We estimated relative gallinule population density at each wetland using habitat area derived from the National Wetlands Inventory (US Fish and Wildlife Service, 2010) and abundance estimates from population surveys (Table 1). Population surveys followed DesRochers *et al.* (2008), using 30 seconds of call playback followed by 1 minute of observation every 20 m around the perimeter of the habitat. Habitat area was calculated as the total area contained by a 2 m buffer around the edges of each pond or impoundment within a wetland.

We qualitatively assessed predation pressure by assigning a rank (0–2) based on the degree of predator control used at a site. Introduced mammalian predators like the Small Indian mongoose (*Herpestes javanicus*) are ubiquitous on O‘ahu and their impacts on native waterbirds are well known and can only be reduced by active predator control (US Fish and Wildlife Service, 2011; Reed *et al.*, 2012). Feral and domestic cats (*Felis catus*), dogs (*Canis lupus familiaris*) and other human-associated animals are also a threat to these birds (US Fish and Wildlife Service, 2011). We assigned a rank of 0 to sites with heavy or constant active predator control, 1 to sites with limited/inconsistent predator control and 2 to sites with no predator control. We defined limited predator control as control that was sporadic or covered <25% of the impoundment habitats in a wetland and heavy predator control as control measures that were constantly implemented and covered >25% of impoundment habitats. We assessed predator control intensity through consultations with land managers.

We also assessed proximity to urban development by calculating the percentage of urban cover (defined as impervious surface) within 1 km of the wetland complex centroid for each sampled location. This was completed using NOAA CCAP dataset from 2005 (NOAA, 2005) in QGIS version 3.6.1 (QGIS Development Team, 2018). Despite their age, these data represent the highest quality and most recent estimates of impervious (urbanized) land cover at an acceptable spatial resolution (30 m) for the island of O‘ahu. Individual wetlands were checked against more recent (ca. 2019) aerial photography from Google Maps to ensure that no significant changes in surrounding urbanized area had occurred prior to analysis. O‘ahu is the most urbanized of the Hawaiian islands, and the majority of recent urban development is currently taking place in the island’s Southwest corner, where no Hawaiian gallinule subpopulations are known.

Finally, we qualitatively ranked wetlands (0–4) for degree of human disturbance based on the number of visitors to each wetland per day. A minimum of 7 days (throughout a normal work week) was spent at each study site during sample collection, with observers in the field from approximately 0700 to 1800 hours. Wetlands were ranked 0 for <1 visitor per day, and 1, 2, 3 and 4, for 1–10, 11–50, 51–100 and >100 visitors per day, respectively.

### Capture and feather collection

At the time of capture, we collected 5–10 contour feathers from the right underwing (flank or side of body) of 144 Hawaiian gallinules for corticosterone analysis. All individuals were outfitted with US Geological Survey (USGS) aluminum bands and a unique combination of plastic color bands for future identification, ensuring that each sampled bird was a unique individual and that individuals were not double counted. We captured these birds using walk-in live traps baited with cracked corn, banana, cat food and other attractive food items. All capture and handling were done under USGS bird banding, US Fish and Wildlife Service endangered species and Hawaii Division of Forestry and Wildlife endangered species permits and using protocols approved by the Tufts University Institutional Animal Care and Use Committee. Morphometric data were collected for a subset of captured animals; body mass was measured using a 1-kg Pesola spring scale and tarsometatarsus length (as the distance from the inner bend of the tibiotarsal articulation to the base of the toes) using a pair of digital calipers. Body condition was estimated by calculating the body mass-to-tarsus ratio. There is currently not a consensus in the literature as to the best metric of body condition (see reviews by Stevenson and Woods, 2006; Schamber *et al.*, 2009); however, mass-to-tarsus ratio has been used in a number of recent papers that show it can relate to adult body fat in birds (e.g. Kraft *et al.*, 2019; Gaiotti *et al.*, 2020). Additionally, it should be noted that the difference between the lightest (275 g) and heaviest (534 g) birds in the dataset is quite large, increasing the statistical power of this metric. These data were collected for 76% of our dataset (34 missing values). We estimated bird age based on facial shield and leg color, as well as plumage according to Fredrickson (1971) and Cramp and Simmons (1980), and included only mature (after second year) birds in our analysis. We determined sex genetically for a subset of our samples (see methods in van Rees *et al.*, 2018b) and opportunistically when copulation was observed (*n* = 55 females, 80 males, 9 unknown). The sex of several birds where sex was determined behaviorally during copulation was confirmed by genetic analysis. In order to ensure the representativeness of samples, for all sites, trapping and sampling efforts continued until at least 15 adult birds were captured and sampled, or until >50% of the resident subpopulation had been sampled. The latter proportion was determined using population surveys conducted according to DesRochers *et al.* (2008); notably, some local subpopulations consisted of <10, and in one case (Honouliuli), 3 individuals. James Campbell National Wildlife Refuge, with an estimated abundance of more than 80 individuals (van Rees and Reed, 2018), was the only exception to this goal.

### Feather corticosterone assays

Flight feather molt in Hawaiian gallinules occurs nearly year round, with little evidence of synchrony between individuals, although all flight feathers are molted simultaneously in the same individual (DesRochers *et al.*, 2009). By contrast, contour feathers are molted singly throughout the year, making the time frame of feather growth and stress hormone deposition unsystematic among feathers and individuals. Consequently, we can be confident that each feather was molted within the previous year, but not a specific period within that year. The contour feathers of any individual thus reflect an effectively random sample of relative blood corticosterone levels for a time period from the minimum time required for feather development up to approximately 1 year earlier. As demonstrated with molecular and mark-recapture evidence (van Rees *et al.*, 2018a, 2018c), Hawaiian gallinules are highly sedentary and movement between habitats is highly unlikely in any given year. This lends strong support to the assumption that an individual feather sample represents an integrated measurement of the stress levels driven by local habitat-level stressors in the habitat or subpopulation in which it was captured. Habitat conditions within individual wetlands or subpopulations are also known to have been relatively constant throughout the study period and several years prior, with hurricanes and extreme weather events being rare on O‘ahu and water levels in wetlands being predictable, consistent and controlled for aesthetic (golf course, botanical garden), agricultural (lotus and shrimp farms) or wildlife management (federal and state refuges) reasons. This high consistency in other habitat variables negates many potential confounding effects upon habitat-level feather stress hormone levels.

Feathers were prepared and assayed following the methods previously detailed (Bortolotti *et al.*, 2008; Lattin *et al.*, 2011), with some modifications. Briefly, prior to measuring the length of each feather, the calamus was removed. The feathers were then cut with scissors into 5 mm^2^ sections into conical Falcon 15 ml tubes. Feather mass was standardized to 0.01 g (±0.002 g), which was approximately four feathers per individual. If only a portion of a feather was used to reach the target mass, the distal section was minced first. The mass of all samples was standardized to 0.01 g in order to improve consistency of results, since past studies have shown that feather corticosterone levels vary with mass, particularly with very small samples (Lattin *et al.*, 2011; Jenni-Eiermann *et al.*, 2015).

All feather samples were randomized prior to assaying. In order to extract steroid hormones from the feathers, 7 ml of methanol was added to each tube. Tubes were then placed in a sonicating water bath for 30 min at room temperature. Samples were maintained in a shaking water bath at 50°C overnight. The following morning, the methanol was separated from the feathers using a vacuum filtration process with #4 Whatman filters. Remaining feather bits, the sample vial and the filter paper were washed twice with an additional 2.5 ml methanol each time. The methanol extracts were then evaporated under nitrogen gas in a 50°C water bath, reconstituted using a Tris HCl buffer and left to stand overnight in a refrigerator.

The samples were run through a standard radioimmunoassay (Wingfield *et al.*, 1992) using a Sigma-Aldrich antibody (C8784; St Louis, MO, USA). The inter-assay coefficient of variation was 16.1% based on both standard corticosterone and feather pools and the intra-assay coefficient of variation 4.2%; three total assays were run. Seven samples were below the limit of detection and therefore were assigned values based on the floor of the assay and the length of feather. The average floor of assays was 0.18 pg/mm. Feather corticosterone was standardized by the length of each feather sample (Jenni-Eiermann *et al.*, 2015).

### Statistical analyses

We analyzed our data using RStudio 3.6.2 (RStudio Team, 2015). We checked the distributions of all explanatory variables visually using histograms. All were normally distributed, but feather corticosterone was log-transformed. First, a univariate linear model was constructed to assess whether sample year impacted feather corticosterone. After concluding it did not have a significant impact (*F*_2,140_ = 0.81, *P* = 0.45), it was removed from future models. To confirm this decision, we created separate univariate models with sample year as a random effect. In the majority of cases, this led to overly complex models with singular fits; thus, our decision to exclude this random effect was supported. Univariate linear models were constructed with feather corticosterone as a response variable and the following as covariates: level of predation control; level of human disturbance; conspecific population density; percent urban cover within 1 km; condition (g body mass/mm tarsus length); body mass; and sex. In the cases of predation control and human disturbance (both ordinal variables), the ‘Anova’ function was used to generate *P* values (car package; Fox and Weisberg, 2011). In all other cases, the ‘summary’ function was used to summarize the models. One combined mixed effects model with all covariates (except mass) was also constructed and model quality was assessed by *R*^2^ (‘r2’ function, sjstats package; Lüdecke, 2019) and *P* values (‘Anova’ function); mass was excluded to avoid potential collinearity with condition. In this global model, site was included as a random effect and the following were included as covariates: level of predation control; level of human disturbance; conspecific population density; percent urban cover within 1 km; condition (g body mass/mm tarsus length); and sex. Multi-collinearity was assessed using the ‘vif’ function (car package; Fox and Weisberg, 2011). Visitation had a generalized variance inflation factor (GVIF) of 13.5, which could indicate collinearity; however, we chose to retain visitation in the model as there is controversy over proper thresholds for GVIF values (O’Brien, 2007) and when it was removed, an overly complex, singularly fit model resulted. We also constructed univariate models to investigate drivers of body conditions. Again, sample year did not have a significant impact (*F*_2,107_ = 0.10, *P* = 0.90). Covariates included site, density, predation control, human disturbance level, sex and percent urban cover within 1 km. Residual plots were visually inspected for normality in all cases; Q-Q plots were normally distributed in all cases.

``R code and data available upon request.''

## Results

Sampled wetlands covered the full range of rankings for level of predator control, human disturbance and percent urban cover (human disturbance: x̅ ± SD = 1.4 ± 0.36; predator control: x̅ ± SD = 1.23 ± 0.73; percent urban cover: x̅ ± SD = 34.71 ± 20.48; Table 1), and population density of Hawaiian gallinules ranged from 0.67 to 34.9 individuals/ha (x̅ ± SD = 9.28 ± 19.15; Table 1). The only covariates that showed any statistically significant correlation were gallinule population density and individual condition, where a statistically significant negative relationship was observed, with individual condition decreasing at higher population densities (β = −0.014 ± 0.006, *P* < 0.035; Fig. 2F. Mass-to-tarsus ratio, our proxy for individual condition, ranged from 4.1 to 8.7 g/mm (x̅ ± SD = 5.74 ± 0.05, *n* = 110).

Feather corticosterone tended to be consistent across all sampled Hawaiian gallinules (x̅ ± SD = 0.85 ± 0.69 pg/mm), and there were no obvious patterns between wetlands (Fig. 1). Once log-transformed, feather corticosterone followed a normal distribution. None of our univariate linear models or our full model showed any statistically significant relationships between feather corticosterone and our wetland- or individual-scale explanatory variables (all *P* > 0.09 in all cases; Fig. 2, Table 2). The multivariate mixed effects model also did not reveal any significant relationships between covariates (*R*^2^_c_ = 0.17, *R*^2^_m_ = 0.04; Condition, β = −0.21 ± 0.14, *P* = 0.13; Density, β = 0.001 ± 0.02, *P* = 0.95; Percent urban cover, β = −0.004 ± 0.009, *P* = 0.67; Visitation-1, β = 0.04 ± 0.48, Visitation-2, β = 0.04 ± 0.43, Visitation-3, β = −0.28 ± 0.58, Visitation-4, β = −0.03 ± 0.66, *P* = 0.99; Sex-male, β = 0.03 ± 0.19; Sex-unknown, β = 0.24 ± 0.43, *P* = 0.85; Predator control-1, β = 0.23 ± 0.58, Predator control-2, β = 0.11 ± 0.70, *P* = 0.91).

**Table 2:** Summary of the univariate linear models constructed to explore predictors of feather corticosterone (log-transformed) and body condition of Hawaiian gallinules. Bolded values indicate statistical significance. Note corticosterone is abbreviated as Cort

Dependent variable	Predictor variable	*F*-statistic	Degrees of freedom	*P* value	Adjust *R*^2^
logCort	Condition	2.27	1, 108	0.14	0.01
	Density	0.06	1, 142	0.80	−0.007
	Human disturbance	2.11	1, 142	0.15	0.008
	Mass	0.76	1, 118	0.39	−0.002
	Percent urban cover	2.94	1, 142	0.09	0.01
	Predation control	0.26	2, 141	0.77	−0.01
	Sex	0.42	2, 141	0.66	−0.008
	Site	1.36	14, 129	0.18	0.03
	Year	0.81	2, 140	0.45	−0.003
Condition	Density	4.77	**1, 108**	**0.03**	0.03
	Human disturbance	1.65	1, 108	0.20	0.006
	Percent urban cover	0.15	1, 108	0.70	−0.008
	Predation control	0.06	2, 107	0.95	−0.02
	Sex	12.98	2, 107	**<0.0001**	0.18
	Site	1.7	14, 95	0.07	0.08
	Year	0.10	2, 107	0.90	−0.02

## Discussion

The purpose of this study was to assess feather corticosterone levels as a bioindicator of Hawaiian gallinule stress physiology across several conservation-relevant ecological gradients: urban–rural, amount of human contact, index of predation risk and conspecific population density. The work was done across the species range on the island of O‘ahu. Interestingly, we found no significant patterns between measured habitat-level (human disturbance, predation control, population density) or individual-level (body condition) characteristics and feather corticosterone (Figs 1 and 2). We were particularly surprised by this result because the range of each of our stressor gradients was extremely high, to the point that achieving greater levels of environmental variation might have been difficult while still keeping the target species present. Additionally, our dataset represents a robust sample of Hawaiian gallinules of various sizes (range, 275–534 g), providing sufficient variation in condition to detect effects of or on condition. Accordingly, the dataset used in this work is highly robust in sample size and in spread of habitat characteristics, providing high power to detect potential effects of stressors at multiple scales upon feather corticosterone. The negative results of this study highlight an important perspective and cause for caution in the use of an emerging technique in conservation biology, as detailed below.

The lack of clear relationship between feather corticosterone and level of predation control (Figs 1A and 2A) was a surprising finding, considering the extremely wide range in predation control conditions sampled in this study. For instance, James Campbell National Wildlife Refuge actively removes and prevents predators with standardized, regularly monitored trapping programs for several species (mongoose, feral cats, predators of adult birds) and active culling of other potential predators like the American bullfrog (*Lithobates catesbianus*) and Black-crowned Night-heron (*Nycticorax nycticorax*), which depredate chicks and juveniles (Byrd and Zeillemaker, 1981; Chang, 1990). At other sites (e.g. Turtle Bay, Olomana) predators including mongoose and feral cats are frequently encountered while sampling gallinules and no efforts are made to control or exclude them from habitats (CBvR, JMR pers. obs.). The large magnitude of variation in predator control efforts—a proxy for predator densities and pressure—across our sample makes it unlikely that insufficient sample variation is driving our observations. This contrasts with previous studies, which have often found that higher levels of predation pressure correlate with elevated baseline corticosterone. For example, it has been shown that when predators are excluded from habitats, song sparrow (*Melospiza melodia*) baseline and stress-induced blood corticosterone decreased (Clinchy *et al.*, 2004), and that that effect is stronger in males than females (Newman *et al.*, 2013). It has also been shown that eiders (*Somateria mollissima*), which nested with greater coverage—and therefore with more protection from predators—tended to have reduced corticosterone stress responses (Jaatinen *et al.*, 2014). Our study suggests that the relationship between predation pressure and corticosterone is not always consistent.

Secondly, we found no relationship between feather corticosterone and level of human disturbance or magnitude of urban cover (Figs 1B and D and Figs 2B and D). Human-altered environments introduce a suite of novel stressors, including altered food resources, habitat fragmentation and traffic noise, all of which can positively or negatively impact survival depending on the species (e.g. Kark *et al.*, 2007; Lowry *et al.*, 2011; Isaksson, 2015). One study found that great tits (*Parus major*) in urban environments have distinct gene expression profiles, particularly for genes associated with the stress response (Watson *et al.*, 2017). Despite these possible underlying patterns of physiological activation, in general there does not appear to be a consistent pattern in how urbanization influences glucocorticoid release (Injaian *et al.*, 2020; see Supplemental Table 1 in Strubbe *et al.*, 2020), and the responses seem to be species-specific (Bonier, 2012). From a behavioral point of view, the lack of detectable change in mean feather corticosterone across a large range of human disturbance may imply that Hawaiian gallinules are urban adapters. Their use of many urban habitats suggests that they can acclimatize readily to human-altered landscapes (Cyr and Romero, 2009); however, predator control is necessary for successful reproduction and local persistence without immigration. Despite the lack of a physiological signal, the individuals in this study did present a range of behaviors in response to humans. Estimated flight initiation distances varied from less than 1 meter at Waimea Valley and Keawawa wetland, both sites with heavy human visitation, to over 30 meters at James Campbell and Pearl Harbor (Honoululi) National Wildlife Refuges, where birds fled and hid at any sign of human presence or activity within line of sight (CBvR, pers. obs.). These qualitative results further suggest that the experience and behavioral adaptation of these birds differ across the various habitats, even though these differences are reflected in behavior, but not feather corticosterone levels.

Thirdly, we found no relationship between feather corticosterone and conspecific population density (Figs 1C and 2C), a proxy for the potential stress of aggressive intraspecific competition for space. The effects of the social environment on HPA axis regulation, particularly in territorial animals, are well documented (reviewed by Creel *et al.*, 2013). In general, density and glucocorticoid levels are positively correlated, although this relationship has been more thoroughly tested in mammals. Hawaiian gallinules exhibit strong intra- and interspecific (with Hawaiian coot, *Fulica alai)* territorial behavior, which often culminates in violent agonistic encounters (CBvR pers. obs; van Rees *et al.*, in press) that can result in injury. The incidences of these interactions are potentially exacerbated in habitats with higher densities of gallinules and coots (van Rees *et al.*, in press). Our observation that Hawaiian gallinules in more densely populated habitats tend to have poorer condition (body mass/tarsus length) supports this notion (Fig. 2F). We believe that this finding is consistent with the idea that gallinules experience different magnitudes of local challenges and that density-dependent dynamics are a powerful factor influencing individual condition and possibly reproductive success and population dynamics. This is of particular importance for Hawaiian waterbirds, which appear to be habitat limited. Since European colonization, an estimated 75% of low elevation coastal wetland on O‘ahu has been lost (van Rees and Reed, 2014), and density-dependent dynamics—specifically hypothesized to result from the impacts of intraspecific agonistic interactions on reproductive success—have been detected at the population and local level in another endangered Hawaiian waterbird (van Rees *et al.*, 2020). We also acknowledge that there are a number of different indices of body condition (see reviews by Stevenson and Woods, 2006; Schamber *et al.*, 2009) and there is no consensus as to which most accurately relates to body fat. In addition to mass-to-tarsus ratio, we also found a significant relationship between mass and density, further supporting our conclusion that Hawaiian gallinules in denser populations are in worse overall condition.

Finally, it is worth noting that our study applies to only half of the Hawaiian gallinule’s global range (the island of O‘ahu), while an equal or greater number of birds resides on Kaua‘i. The total variance in environmental stressors on Kaua‘i is likely to be lower than that on O‘ahu for several reasons, including much lower levels of landscape development, human population densities and wetland loss (van Rees and Reed, 2014), and no established population of Small Indian mongoose. By contrast, gallinule population densities in the primary population stronghold on Kaua‘i, Hanalei National Wildlife Refuge, may be at the upper end or exceeding the range tested in this study, and additional stressors of disease (occasional outbreaks of avian botulism) and flooding events that destroy nests are prominent threats not present on O‘ahu and not tested in this study (US Fish and Wildlife Service, 2011). Parallel work on the stress physiology, movement, population connectivity and population viability (as in van Rees *et al.*, 2018a, 2018b, and van Rees and Reed, 2018, and this study) is needed for the population on Kaua‘i , which is comparably understudied and projected to be valuable to the subspecies’ survival under future sea level rise. It is probable that density-dependent impacts on body condition as detected in this study, and potential effects on reproduction may be of greater concern in high-density habitats on Kaua‘i, but the management implications of this work are the same for both islands in the subspecies’ current distribution.

## Conclusions

This study provides strong evidence that physiological metrics of stress do not always correlate with habitat characteristics commonly used in applied conservation, even across large ranges of habitat condition. Several key advantages of this study enabled a more thorough testing of the efficacy of feather corticosterone as a measure of habitat quality in an endangered subspecies. Firstly, our study system was geographically expansive, including 13 sampled sites representing all known breeding habitats on O‘ahu, which makes up 50% of the subspecies’ current global distribution. Secondly, sampled habitats were diverse in levels of predation control, human disturbance, urban development and conspecific population density (Table 1). This variation produces an expansive range in stressor exposure (in some cases across orders of magnitude), both in mechanism (e.g. individual body condition vs. predation pressure vs. human disturbance) and magnitude. Additionally, we were able to sample a relatively large number of Hawaiian gallinules at many discrete, replicated sampling sites. Our study also focused on a cryptic and poorly understood species of conservation concern, for which there is a deficit of physiological data.

The distinct advantages of this dataset add credence to our findings, which suggest that feather corticosterone may not always be a good indicator of other individual or habitat-level metrics that are of interest for the management and conservation of threatened and endangered bird species. Consequently, relying on feather corticosterone levels as a bioindicator should not be done for any species until validation work has been performed. As previous reviews have suggested, assessment of glucocorticoids cannot be useful in conservation-related decisions if concentrations do not vary *even* when we know that habitat quality varies (Busch and Hayward, 2009).

## Funding

Funding for sample collection was provided by the Tufts Institute of the Environment, Tufts Graduate School of Arts and Sciences, Nuttall Ornithological Club, USGS Alaska Science Center, USFWS, Wilson Ornithological Society, Sigma Xi Grants-in-Aid of Research, Disney Conservation Fund and Tufts Water Diplomacy IGERT (NSF 0966093). Funding for feather corticosterone analysis was provided by NSF IOS-1655269 (to L.M.R.).
